# Optimizing microtubule arrangements for rapid cargo capture

**DOI:** 10.1016/j.bpj.2021.10.020

**Published:** 2021-10-21

**Authors:** Saurabh S. Mogre, Jenna R. Christensen, Samara L. Reck-Peterson, Elena F. Koslover

**Affiliations:** 1 Department of Physics, University of California San Diego, La Jolla, California; 2 Department of Cellular and Molecular Medicine, University of California San Diego, La Jolla, California; 3 Division of Biological Sciences, Cell and Developmental Biology Section, University of California San Diego, La Jolla, California; 4 Howard Hughes Medical Institute, Chevy Chase, Maryland

## Abstract

Cellular functions such as autophagy, cell signaling, and vesicular trafficking involve the retrograde transport of motor-driven cargo along microtubules. Typically, newly formed cargo engages in slow undirected movement from its point of origin before attaching to a microtubule. In some cell types, cargo destined for delivery to the perinuclear region relies on capture at dynein-enriched loading zones located near microtubule plus ends. Such systems include extended cell regions of neurites and fungal hyphae, where the efficiency of the initial diffusive loading process depends on the axial distribution of microtubule plus ends relative to the initial cargo position. We use analytic mean first-passage time calculations and numerical simulations to model diffusive capture processes in tubular cells, exploring how the spatial arrangement of microtubule plus ends affects the efficiency of retrograde cargo transport. Our model delineates the key features of optimal microtubule arrangements that minimize mean cargo capture times. Namely, we show that configurations with a single microtubule plus end abutting the distal tip and broadly distributed other plus ends allow for efficient capture in a variety of different scenarios for retrograde transport. Live-cell imaging of microtubule plus ends in *Aspergillus nidulans* hyphae indicates that their distributions exhibit these optimal qualitative features. Our results highlight important coupling effects between the distribution of microtubule tips and retrograde cargo transport, providing guiding principles for the spatial arrangement of microtubules within tubular cell regions.

## Significance

Tubular cell projections such as neuronal axons and fungal hyphae require a flux of cellular components delivered from the periphery to the cell body via retrograde transport driven by dynein motors along polarized parallel microtubule tracks. Some newly formed cargos are loaded at specialized dynein-rich capture regions near microtubule plus ends before initiating retrograde transport. For such systems, the relationship between the spatial arrangement of microtubules and the efficiency of transport initiation remains poorly understood. In this work, we develop a quantitative picture of the cargo capture process by a variety of microtubule configurations and relate our findings to observations from *Aspergillus nidulans* fungal hyphae.

## Introduction

Microtubules form an essential component of the intracellular transport system, allowing for long-distance distribution and delivery of components driven by kinesin and dynein motors. In eukaryotic cells, microtubules are organized in a wide variety of arrangements depending on cellular geometry and specific biological transport objectives (1,2). These architectures range from centrally anchored radial arrays to swirling or planar-polarized structures nucleated at the cell periphery to parallel structures in the narrow cylindrical domains of neuronal projections and fungal hyphae (3). The stark variation in cytoskeletal organization across different cell types raises a fundamental question regarding how the arrangement of microtubules affects cargo transport functionality. Furthermore, pharmacological modulation of cytoskeletal architecture by stabilization of dynamic microtubules has been proposed as a potential intervention to reduce transport deficits associated with neurological injury and disease (4).

Many studies have sought to relate the efficiency of cargo transport with cytoskeletal filament arrangements in various contexts. For disordered networks, the dependence of cargo delivery time on filament polarity, bundling, length, orientation, and local density has been established via continuum models and simulations of explicit network architectures (5, 6, 7, 8, 9, 10). Cellular-scale cargo distribution in these models generally relies on multimodal transport, incorporating processive runs whose direction is determined by the microtubule arrangement interspersed with pauses or diffusive phases that allow transition between microtubules (11,12). The microtubule architecture thus modulates transport efficiency both by directing processive motion and by determining the rate of capture for cargo in the passive state.

One biologically important objective for intracellular transport is the capture of newly formed cargo and its delivery toward the perinuclear region. Cargos destined for the nucleus often exhibit directed retrograde motion or bidirectional motion that is substantially biased in the retrograde direction. Such cargo includes signaling endosomes (13,14) or autophagosomes (15) formed at distal regions or COPII-coated vesicles that bud from the endoplasmic reticulum throughout the cell (16). Because directed motor-driven transport is much faster than diffusion of vesicular organelles, the initial step of cargo capture can play an important role in determining the overall timescale of delivery toward the nucleus.

Cells with long tubular projections, such as neurons and fungal hyphae, provide a particularly convenient model system for retrograde cargo transport. In neuronal axons, microtubules are highly polarized, with their plus ends pointing toward the distal tip (17). Similar plus-end-out polarization is observed in the distal segment of multinucleated hyphae for fungi such as *Aspergillus nidulans* and *Ustilago maydis* (18). Here, we consider the efficiency of cargo capture for transport toward the cell body in these tubular model systems.

Because these geometries are much longer than they are wide, the axial distribution of cargo capture positions becomes particularly important. Given the typical diffusivity of vesicular organelles on the order of *D* ≈ 0.01 *μ*m^2^/s, it should take on the order of 1 min for cargo to explore the radial cross section of an axon or hypha with radius approximately 1 *μ*m. By contrast, the time to reach the cell body via pure diffusive transport would range from hours (for a 10 *μ*m hyphal tip) to years (in a millimeter-long axon). Cells rely on processive retrograde transport to replace these unreasonably long timescales with a much more rapid directed velocity on the order of 1 *μ*m/s.

In animal and fungal cells, processive retrograde transport is carried out primarily by cytoplasmic dynein-1 motors that carry cargo toward anchored microtubule minus ends. In some cell types, dynein accumulates near the tips of growing microtubules (plus ends) and forms enriched pools referred to as “comets” that can act as localized capture regions for cargo (19, 20, 21, 22, 23). The placement of comets can be controlled by varying microtubule length (or nucleation sites in axons), and their positioning in relation to where cargo is formed can determine the diffusive search time before initiation of active transport.

We consider the process of cargo binding to a dynein comet and look for possible arrangements of microtubule plus ends along the axial direction that facilitate this process for various positions of cargo entry. Our focus is on the initial cargo capture process, with the processive retrograde motion assumed to be relatively fast regardless of the microtubule arrangement.

We begin by examining simplified microtubule arrangements that capture cargo entering at the distal tip of a cell. Using an analytic one-dimensional model to represent the tubular geometry, we calculate the capture time, defined as the mean first-passage time (MFPT), to encounter the capture zone at a microtubule plus end. We derive the conditions for which the MFPT is minimized and validate the one-dimensional approximation using three-dimensional Brownian dynamics simulations. The analysis is then extended to include broadly distributed randomized microtubule configurations evaluated for efficiency of cargo capture with different initial distributions. General features are established for microtubule arrangements that allow efficient capture for both distally and uniformly derived cargos. A minimal model of microtubule dynamics highlights how such optimal arrangements may be obtained by tuning microtubule catastrophe rates. Additional results are provided to establish that the optimal configuration remains efficient even when cargo can be captured throughout the microtubules and even when the retrograde transport time itself is explicitly taken into account. Finally, we quantify live-cell images of *A. nidulans* fungal hyphae to demonstrate that observed microtubule distributions in hyphal tips exhibit the general features identified for optimal arrangements.

## Materials and methods

Experimental methods for imaging microtubules in *A. nidulans* hyphae are provided in the Supporting materials and methods, Section S9. The development and implementation of the mathematical model and computational simulations are described below.

### Model development assumptions

We briefly summarize the fundamental underlying assumptions that motivated the construction of our mathematical models.

1) The cargo motion before capture by a microtubule is assumed to be diffusive in nature. In particular, we assume cargos detach from the plasma membrane before capture at the microtubule tips. Apparently diffusive trajectories in the cytoplasm have been observed for fungal peroxisomes (24), as well as virus-laden endosomal particles and lysosomes in mammalian cells (25,26) and endocytic particles in yeast (27). In some cases, vesicular particles undergo subdiffusive rather than diffusive motion (28,29). In many others, the apparent random walk behavior arises not from thermal Brownian motion, but rather from spatially dispersed active forces from actomyosin contraction (30,31) or hydrodynamic entrainment by passing motor-driven organelles (24). Newly formed organelles such as endosomes may exhibit short-range motion along the cortical actin cytoskeleton shortly after release from the membrane (27). For simplicity, we subsume all these behaviors in an effectively diffusive model. To plug in a concrete diffusivity, we use the value of *D* ≈ 0.01 *μ*m^2^/s measured for fungal peroxisomes (24), but other effective diffusivities could easily be utilized in the context of this model.
2) The cellular domain is assumed to be a cylinder that is much longer than it is wide. This assumption is relevant for, e.g., fungal hyphae tips (1 *μ*m wide and 5–40 *μ*m long (24,32)) and neuronal axons (a few micrometers across (33), with lengths ranging from hundreds of micrometers to over a meter (34)).
3) We assume cargo capture occurs primarily at microtubule plus ends, which serve as a site for accumulation of dynein motors. This assumption is in concert with prior models of search and capture of cellular targets by proteins bound at the microtubule plus ends (35, 36, 37). In particular, the accumulation of dynein motors in comet-like regions at the microtubule tips is thought to lead to enhanced capture at the plus end for endosomes and other organelles (20,21,23). We also briefly explore the opposite extreme of capture along the full microtubule when establishing the optimal microtubule architectures for rapid capture. In the Supporting materials and methods, Section S8, we examine this assumption explicitly by considering capture regions of different length.
4) We focus on the initial capture process of cargo onto microtubules, assuming that subsequent transport proceeds in a rapid, directed fashion in the retrograde direction toward the cell body. Although many types of cargo are known to exhibit bidirectional movement (20,38), we focus specifically on cargo (such as neuronal autophagosomes (39) and signaling endosomes (14)) that are primarily retrograde. This simplifying assumption also holds for cargo that move bidirectionally with a retrograde bias. Thus, the capture times obtained here provide a lower limit, to which should be added an additional time for retrograde transit to the cell body. From any given position along the domain, retrograde transport tends to be much faster than diffusive transport, implying that optimal microtubule structures should be determined primarily by the initial capture process. This assumption is examined further in Supporting materials and methods, Section S7, in which the additional transit time to the cell body is explicitly incorporated.
5) For clarity of visualization and discussion, we assume microtubules are nucleated near the cell body so that the position of plus-end tips is equivalent to microtubule length. This assumption is valid for fungal hyphal tips in the region past the last nucleus (40). In neuronal axons, shorter microtubules are nucleated in a staggered fashion throughout the domain. However, retrograde-moving cargo tends to step past the microtubule minus ends onto the next microtubule segment with only short (few-second) pauses that imply the cargo is unlikely to dissociate fully into a diffusive state (41). Even with these tiled microtubule arrangements, the retrograde cargo can exhibit effectively processive motion. Thus, we focus on the positions of microtubule plus ends, regardless of their actual length, for modeling cargo capture.
6) To highlight the role of steady-state cytoskeletal architecture, we focus primarily on a model with stationary microtubules. This assumption is then relaxed to incorporate a basic model of microtubule dynamics, reminiscent of prior work on search and capture by microtubule plus ends (35,37).

### Simplified model system for cargo capture

To explore the role of microtubule configurations on cargo capture in a narrow cellular domain, we leverage both an analytically tractable one-dimensional model and three-dimensional Brownian dynamics simulations for cargo motion in a tube.

**Figure 1:**
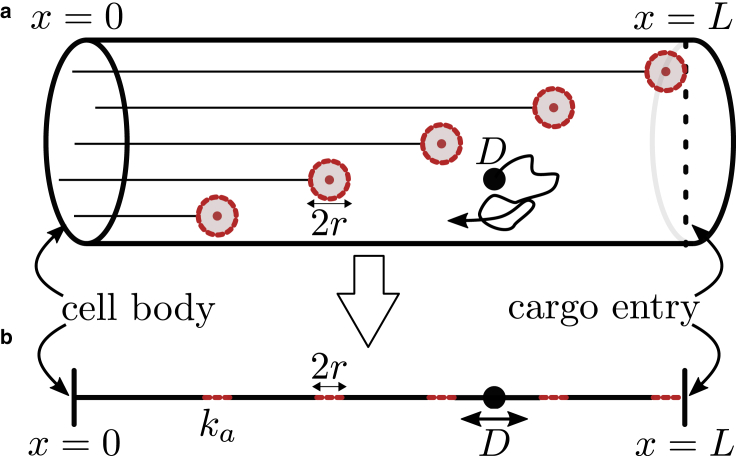
Schematic for cargo capture at microtubule plus ends in tubular cells. (*a*) A depiction of a simplified microtubule arrangement in a tubular cell. Cargo enters at *x* = *L* and diffuses with diffusion coefficient *D*. Cargo is captured at microtubule plus ends depicted as red circles. (*b*) Schematic of the equivalent one-dimensional model. A capture rate *k*_*a*_ is introduced to account for the time spent by the cargo diffusing radially at the microtubule plus-end axial location. To see this figure in color, go online.

We consider a tubular domain of length *L* and radius *R*, with *x* = 0 denoting the cell body and *x* = *L* corresponding to the distal end of the cell (Fig. 1 *a*). We set *R* = 1 *μ*m as appropriate for both neuronal axons (33) and fungal hyphae (24,32). The cargo is modeled as a diffusive particle that either enters the cell at the distal tip or starts uniformly distributed throughout the tube. Cargo diffusivity is set to *D* = 0.01 *μ*m^2^/s, in accordance with prior measurements for vesicular cargo in fungal hyphae (24). Microtubules are treated as straight axial filaments, assumed to be scattered uniformly throughout the radial cross section of the domain. The filaments are assumed to be polarized with their minus ends at the cell body (*x* = 0) and their plus ends distributed at different axial positions.

Cargo is assumed to load onto a microtubule plus end (representing a dynein-enriched comet region) instantaneously upon entering within the capture range (*r* = 0.2 *μ*m) of the microtubule tip. The range *r* is taken to represent a typical contact range from an organelle to a point-like microtubule plus end. The effect of longer comet lengths is explored further in the Supporting materials and methods, Section S8. Throughout the text, we refer to the engagement of cargo to a microtubule via dynein comets as the “cargo capture” process. If the cargo reaches the proximal end of the domain without interacting with a plus end, it is assumed to have been absorbed at the cell body.

### Analytic one-dimensional model

For very narrow domains (*R* ≪ *L*), the simple model described above can be mapped to an effectively one-dimensional system, as illustrated in Fig. 1 *b*. The axial positions of cargo and microtubules are projected onto the axis of the cell, represented by a linear segment of length 0 ≤ *x* ≤ *L*. Cargo can be captured while diffusing within absorbing intervals in the domain, with the rate of absorption determined by the particular arrangement of microtubules.

Plus ends are denoted as discrete intervals of width 2*r* = 0.4 *μ*m, placed at specific axial positions. In an interval corresponding to one microtubule end, the capture rate is set to *k*_*a*_, representing the rate of encountering the microtubule by diffusion across the radial cross section. The value of *k*_*a*_ is estimated by computing the MFPT in a reflecting cylinder of radius *R* to a central absorbing cylinder of radius *r* (42), according to

$$k_{a}=\frac{8DR^{2}}{4r^{2}R^{2}-r^{4}-3R^{4}-4R^{4}\text{ln}\left(r/R\right)}\approx 0.02{\text{s}}^{-1}$$

for the estimated parameters *R* ≈ 1 *μ*m, *r* ≈ 0.2 *μ*m, and *D* ≈ 0.01 *μ*m^2^/s. For microtubule configurations with multiple nearby plus ends, the absorption rate is assumed to scale linearly with the number of plus ends whose capture range overlaps in a given interval. Under this set of assumptions, a particular arrangement of microtubules can be represented by a series of linear intervals with varying absorption rates that are integer multiples of *k*_*a*_. Cargo capture is then represented by a one-dimensional diffusive process in a domain with a reflective boundary at *x* = *L* (cell tip), absorptive boundary at *x* = 0 (cell body), and discrete partially absorbing intervals distributed throughout its length. The MFPT to capture for this process can be obtained analytically by considering all possible paths of the cargo between the different absorbing intervals. Ref (43) describes a propagator-based approach for computing the MFPT to capture a diffusing particle on a network with heterogeneous absorption rates on individual edges. The linear model described here serves as a specialized case of such a network. Details of the derivation for the linear model are provided in the Supporting materials and methods, Section S1.

### Three-dimensional simulations for capture dynamics

To validate the approximate one-dimensional model, we also carry out three-dimensional Brownian dynamics simulations of cargo capture by microtubule tips, directly reproducing the cylindrical system illustrated in Fig. 1 *a*. The simulations assume a domain of length *L* = 10 *μ*m and radius *R* = 1 *μ*m, reflecting the relevant regime for hyphal tips (region past the last nucleus) in *A. nidulans* fungal hyphae. Microtubules are modeled as parallel straight lines nucleating at the proximal end of the domain and are distributed randomly over the cross section. Diffusing cargos are assumed to be instantaneously captured when approaching within a distance of 0.2 *μ*m from the microtubule plus ends. For a given axial configuration of microtubule ends, the MFPT is computed by averaging over 1000 independent simulations, each sampling a different radial distribution of microtubule positions.

When incorporating microtubule dynamics (in Fig. 6), we turn to a minimal model involving microtubule growth and catastrophe. Microtubules are allowed to grow at a speed *v*_*g*_ = 0.18 *μ*m/s, corresponding to typical speeds measured in the hyphae of the fungus *U. maydis* (44), which displays similar geometry and transport dynamics to *A. nidulans*. A growing microtubule that reaches the distal tip of the cell is assumed to remain paused at that location. Both growing and paused microtubules can enter the shrinking state with a catastrophe rate *k*_cat_. We assume that the cargo capture ability of microtubule plus ends (e.g., presence of dynein comets) is lost upon catastrophe so that there is no capture while in the shrinking state. The number of microtubules in the model (*n*_MT_) refers specifically to capture-capable microtubules. Consequently, microtubules that undergo catastrophe instantaneously disappear, and a new zero-length microtubule in the growing state appears in its place to maintain a constant number of capture-capable regions in the cell.

Based on this model, the steady-state density of growing microtubule plus ends (*P*(*x*)) and the number of paused microtubules at the distal tip (*N*_end_) are given by

(1) $$P\left(x\right)=n_{\text{MT}}\left(\frac{k_{\text{cat}}}{v_{g}}\right)e^{-k_{\text{cat}}x/v_{g}}$$

and

(2) $$N_{\text{end}}=n_{\text{MT}}e^{-k_{\text{cat}}L/v_{g}}$$

The derivation for these expressions is provided in the Supporting materials and methods, Section S2.

Initial microtubule lengths are drawn from this steady-state distribution, and microtubules are allowed to grow and shrink according to the described dynamics. Cargo capture at microtubule tips is simulated using the same process as described for static microtubules. For a given catastrophe rate, we carry out 1000 independent simulation runs, each starting with a different initial configuration of microtubule ends (uniformly sampled in the radial dimension and sampled from Eqs. 1 and 2 in the axial dimension). All simulations are carried out using custom-built code in Fortran 90, parallelized on the Open Science Grid (45,46). Code for both simulations and analytical calculations with the one-dimensional model is provided at https://github.com/lenafabr/transportSimCyl.

A table of the main model parameters is provided in the Supporting materials and methods, Section S3.

### Minimal-distance metric to quantify clustering of capture regions

To quantify the axial dispersion or clustering of capture regions (i.e., microtubule plus ends), we define a “minimal-distance” metric for a configuration of *n*_MT_ points on an interval. Namely, this metric measures the average distance between a uniformly distributed probe and its nearest point in the configuration.

The configuration is described by points *x*_*i*_ ∈ [0, *L*], with 1 ≤ *i* ≤ *n*_MT_. An additional point *x*_0_ = 0 is included to represent absorption at the cell body. The closest capture region for a random number *u* distributed uniformly between 0 and *L* is located at *x*_*i*_ if *u* ∈ (*y*_*i*_, *y*_*i* + 1_), where *y*_*i*_ are the midpoints between consecutive absorbing points (*y*_*i*_ = (*x*_*i*_ _− 1_ + *x*_*i*_)/2, 1 ≤ *i* ≤ *n*_MT_). Endpoints of the domain are denoted by *y*_0_ = 0 and $y_{n_{\text{MT}}+1}$ = *L*, respectively. The average distance between the uniformly distributed probe *u* and its nearest absorbing region is then given by

(3) $$\bar{x}=\frac{1}{L}\;\sum_{i=0}^{n_{\text{MT}}}\;\left(\overset{y_{i+1}}{\underset{y_{i}}{\int }}\left|u-x_{i}\right|\;du\right)=\frac{3x_{n_{\text{MT}}}^{2}}{4L}-x_{n_{\text{MT}}}+\frac{L}{2}-\frac{1}{2L}\sum_{i=1}^{n_{\text{MT}}-1}x_{i}\left(x_{i+1}-x_{i}\right)$$

We use *d* = $\bar{x}$/*L* as the clustering metric throughout the text. Smaller values of *d* correspond to well-dispersed microtubule plus ends, with a minimal value of 1/(4*n*_MT_ + 2) for the configuration in which consecutive points are equally spaced. Larger values indicate clustering of microtubule plus ends along the axis, with a value of *d* = 0.25 for the configuration with all plus ends at the distal tip.

## Results and discussion

### Separation of microtubule ends for distal capture

We first consider the problem of optimizing the axial distribution of a limited number of capture regions (e.g., dynein comets at microtubule plus ends) for rapid capture of diffusive cargo entering at the distal tip of a tubular cell. To begin with, we consider two extreme arrangements of microtubule plus ends. On the one hand, clustering plus ends near the distal tip will enable distally produced cargos to quickly encounter and bind to the microtubule. On the other hand, any cargo that diffuses past the clustered plus ends may then embark on very long trajectories down the tube, resulting in a long-tailed distribution of capture times. In general, a diffusive particle that starts at distance *x*_0_ from one absorbing end of a domain of length *L* will have a MFPT of *τ* = *x*_0_(*L* − *x*_0_)/(2*D*) to reach the ends, a quantity that approaches infinity as the domain becomes infinitely long. By contrast, scattering capture regions broadly throughout the domain ensures a uniform availability of capture regions and precludes very long trajectories before capture. However, if the number of microtubule tips is fixed, such a broad distribution results in a lower density near the distal origin of the particles and forces each one to diffuse further along the axis before encountering a tip. To quantify this tradeoff, we compute the MFPT to capture distally produced diffusing cargo for different spacings of microtubule ends away from the distal tip of the cell.

Specifically, we focus on regularly spaced configurations to explore two key parameters that play a role in cargo capture. First, the number of microtubules (*n*_MT_) determines the number of capture regions that a cargo can attach to, with a higher quantity generally corresponding to faster capture. Second, the axial separation (*s*) of consecutive microtubule ends tunes the breadth of their distribution away from the point of cargo entry. Unevenly scattered microtubule end positions are discussed in subsequent sections.

**Figure 2:**
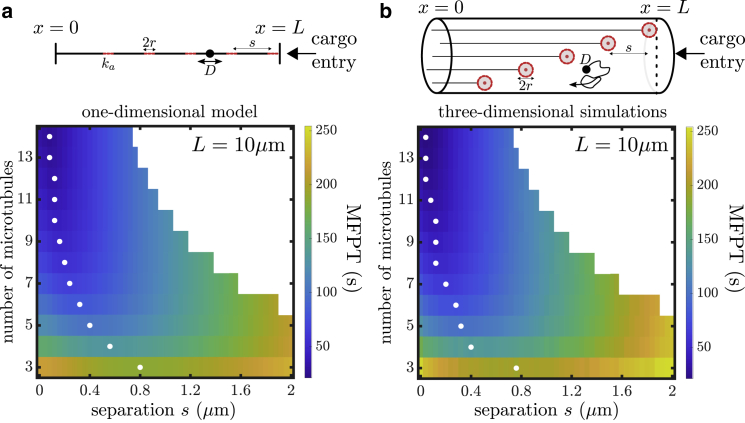
Cargo capture times for axially separated microtubules. (*a*) MFPT as a function of number of microtubules and separation (*s*) between consecutive microtubules for the effective one-dimensional model. The longest microtubule extends from the cell body at *x* = 0 to the cell tip at *x* = *L*, which serves as the point of cargo entry. Subsequent microtubules are axially separated by a distance *s*. White dots mark the separation distance *s* that gives the minimal MFPT for a given number of microtubules. (*b*) Analogous plot for the three-dimensional model of a tubular domain. To see this figure in color, go online.

The geometric parameters of the model reflect a typical hyphal tip (beyond the last nucleus) of the fungus *A. nidulans*, which serves as a convenient model system owing to its neuron-like geometry and genetic tractability. Model construction details are provided in the Materials and methods. An important advantage of a narrow tubular geometry is that it can be modeled analytically as an approximately one-dimensional system. Because the length of our domain is typically much larger than the radius, a simplified model that represents the tube as a line with localized binding regions can encompass the overall behavior of the capture process. The one-dimensional approximation (Fig. 2 *a*, *top panel*) represents the microtubule tips as short intervals with a finite capture rate within each interval that encompasses the rate of radially encountering the microtubule while within that slice of the domain. The average time to capture includes trajectories that pass through multiple capture regions until successfully undergoing capture within one of them. The MFPT of this process can be computed using a previously developed method for reaction rates on heterogeneous tubular networks (43), as described in the Supporting materials and methods, Section S1. Fig. 2 *a* shows a plot of the MFPT versus the number of microtubules and the separation between consecutive microtubule ends.

To verify the validity of the approximate one-dimensional model, we compare our results to three-dimensional Brownian dynamics simulations that encompass the full model system with a tubular domain and spherical capture regions of radius *r* representing the microtubule ends. Details of the three-dimensional model are provided in the Materials and methods. As shown in Fig. 2, the one-dimensional analytic calculations and three-dimensional simulations give nearly identical results for the MFPT. The mean relative error in the MFPT between the one-dimensional and three-dimensional approaches is ∼6.6%. This close correspondence establishes the robustness of the approximate one-dimensional model for representing the narrow tubular geometry. We proceed to employ the one-dimensional model for the remainder of the calculations discussed below.

Interestingly, the MFPT to capture shows nonmonotonic behavior as separation *s* is increased from 0, reaching a minimum at an intermediate separation distance between microtubule ends. The existence of the minimum is a consequence of the competition between capturing cargos quickly near the entry point and extending the overall capture region for cargos that might evade the initial cluster. As the number of microtubules (i.e.: the overall capture capacity) is increased, the optimal separation decreases. This follows from the fact that fewer cargos can escape the initial capture near the tip when a large number of microtubules are present. The optimal separation ranges between ∼0.01 *μ*m for 14 microtubules to ∼0.8 *μ*m for three microtubules. Converting the optimal separation to the overall distance over which plus ends are scattered, the results indicate that it is optimal to distribute plus ends over a distance of ∼1.4–2 *μ*m from the cell tip for a 10 *μ*m cell.

The existence of an optimal separation distance for a given number of microtubules highlights the benefit of scattering capture sites for cargo generated at the cell tip. Intuitively, scattered configurations of microtubule ends are more effective in that they are able to capture cargo that diffuses past the distal region, precluding very long trajectories that explore a large fraction of the domain before returning for capture. Because the MFPT between two absorbing boundaries scales in proportion to the domain length, we would thus expect the optimal microtubule end separation to be larger for longer domains. We therefore proceed to explore the effect of domain length on the optimal microtubule distribution.

**Figure 3:**
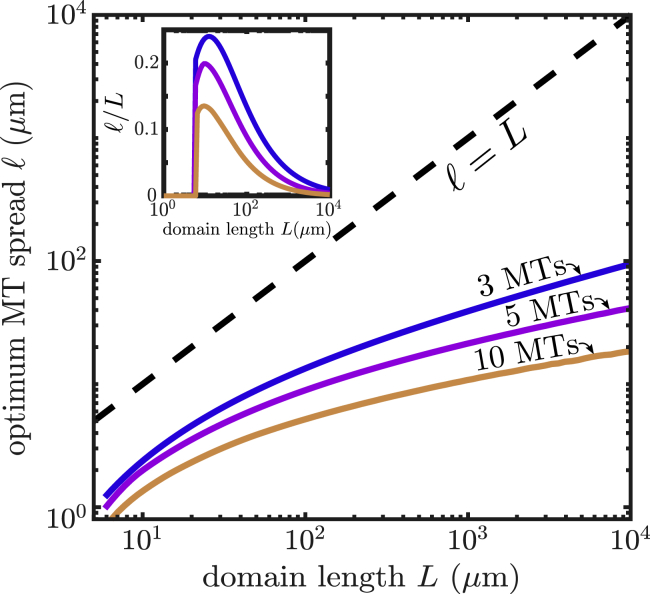
Effect of cell length on microtubule (MT) arrangement. The optimal total spread of microtubule plus ends ($\ell =sn_{\mathrm{MT}}$ ) is plotted versus the length of the cellular domain (*L*) for cargos entering at the distal tip and captured by microtubule plus ends. Inset shows the fraction of the entire domain length over which the plus ends should be spread. To see this figure in color, go online.

Although many tubular cell projections exhibit a similar width of 1–2 *μ*m, the length from the distal tip to the nearest nuclear region can vary widely. In fungal hyphae, the distance from the hyphal tip to the first nucleus can range from a few to tens of microns in length (24,32). Neuronal axon lengths can vary from hundreds of micrometers up to a meter long (34). In Fig. 3 *a*, we compute the optimal separation between microtubule ends for cylindrical domains of different length, where the domain length represents the axial distance from the tip to the nearest nucleus.

As expected, the optimal separation increases for longer domain lengths. However, the rate of increase is distinctly sublinear with *L*. This effect arises because as the region containing the microtubule ends becomes longer, it is increasingly likely that the cargo is captured before leaving to explore the rest of the domain. Because this initial capture process is independent of the domain length, the dependence on *L* becomes increasingly shallower as the microtubule ends are more spread out. Consequently, for long tip-to-nucleus distances, an optimal arrangement of microtubule ends concentrates them over a small fraction of this distance. Cell projections of length 10–20 *μ*m require the widest relative separation of capture regions (Fig. 3 *a*, *inset*), scattering the microtubule ends over ∼15–25% of the domain.

The scattering of microtubule ends engenders a tradeoff between rapidly capturing cargo at its point of entry and minimizing search time for cargo that wanders within the cell. The optimal distribution therefore depends on the location within the cell where cargo first becomes capable of interacting with the microtubule plus ends. In Supporting materials and methods, Section S4, we show how the optimal plus-end separation varies for cargo that must first undergo a maturation process before becoming available for capture. Examples of organelle maturation include neuronal autophagosomes, which may require fusion with other organelles before engaging in retrograde transport (39,47,48). Maturation times above a few minutes allow the cargo to diffuse a substantial distance away from the distal tip so that optimal microtubule plus-end separations become larger.

Varying the maturation rate effectively tunes the initial distribution of capture-ready cargo. The increased optimal separation of capture zones underscores the importance of initial cargo distribution in determining the most efficient arrangement of microtubules. Although there is still a tradeoff between clustered and dispersed microtubule plus ends, matching the location of capture regions to the starting distribution of the cargo leads to more efficient capture. Indeed, a more general treatment would account for various initial cargo distributions. These can be incorporated in the model as initial conditions ranging between two extremes: cargo entering at the cell tip or cargo being distributed uniformly within the cell.

### Optimal microtubule configurations for multiple capture conditions

In the previous section, we focused on cargo produced at the distal tip and loaded onto microtubules only within a 200 nm contact radius of the plus end. However, both of these assumptions do not necessarily hold for all retrograde transport systems. For example, although the distal tips of hyphae are the most endocytically active (49), some endosomes may be produced elsewhere along the membrane. Other organelles, such as peroxisomes, may bud from the endoplasmic reticulum all along the hyphal length. We therefore consider for comparison the extreme case of cargo produced uniformly throughout the extended cell region. Furthermore, dynein comets exhibit a gradual decrease in density over a micrometer length scale (20), so capture may not be limited to such a short range of the microtubule plus end. In this section, we explore the overall features of optimal microtubule configurations for retrograde transport initiation in a variety of cargo production and capture conditions.

**Figure 4:**
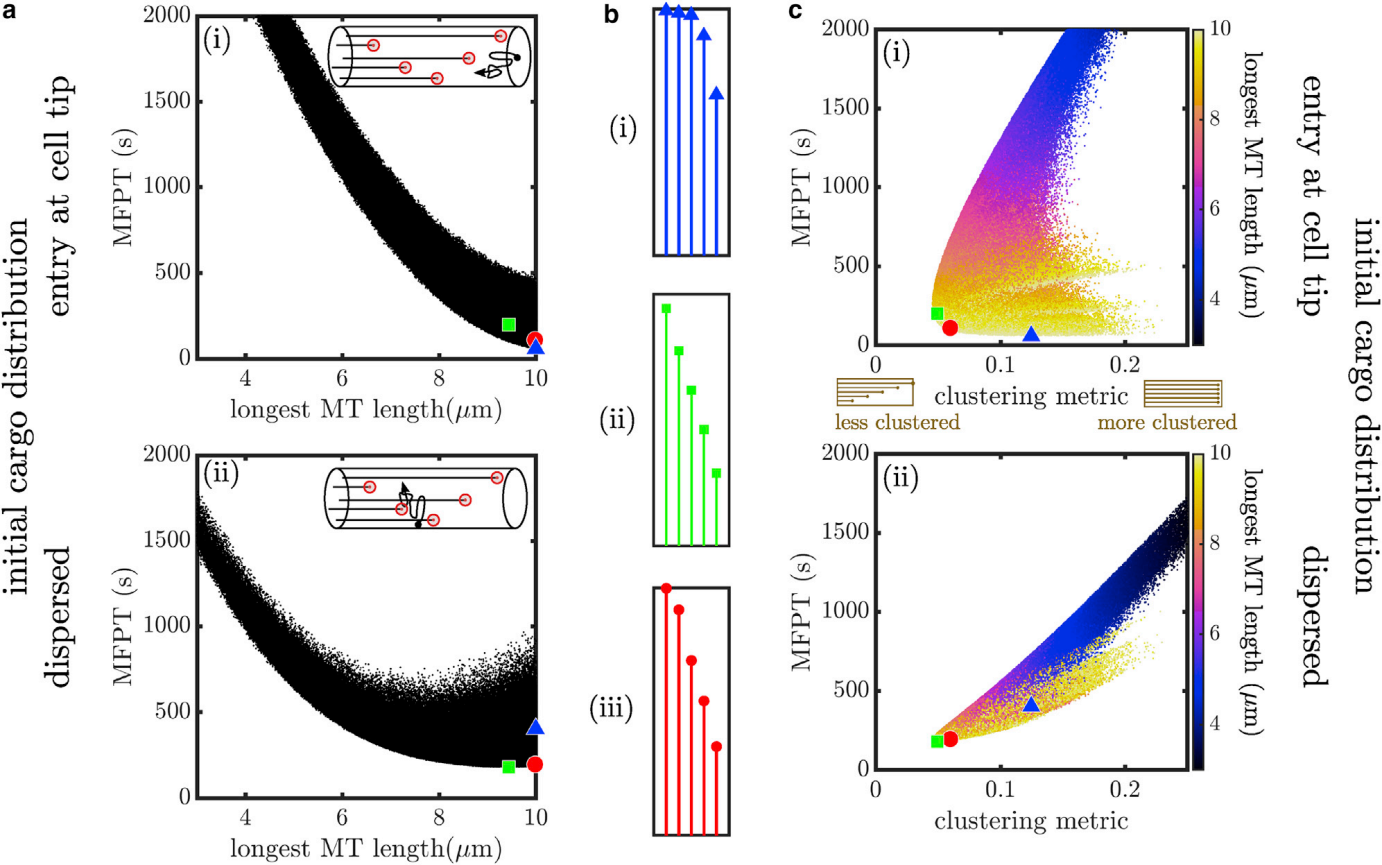
MFPT for random microtubule configurations. (*a*) Scatter plots showing the MFPT to capture at microtubule plus ends versus the length of the longest microtubule (MT) for 10^6^ randomly sampled configurations with five microtubules each in a domain of length 10 *μ*m. (*i*) Cargos start at the cell tip. (*ii*) Cargos start uniformly. Blue triangle indicates the overall fastest configuration for (*i*). Green square indicates the overall fastest configuration for (*ii*). Red circle denotes a configuration that falls within the lowest 3% of MFPTs for all capture conditions studied, including results shown in this figure and in Fig. 5. (*b*) Microtubule configurations corresponding to the (*i*) blue triangle, (*ii*) green square, and (*iii*) red circle in (*a*). (*c*) Scatter plots showing the MFPT plotted against a clustering metric for the randomly sampled configurations, with color indicating longest microtubule length for each configuration. (*i*) Cargos start at cell tip. (*ii*) Cargos start uniformly. Blue triangle, green square, and red circle denote configurations illustrated in (*b*). To see this figure in color, go online.

We generate 10^6^ random configurations of five microtubules, with plus-end positions selected uniformly at random across the domain. The number of microtubules was chosen to be relevant for the tip region of *A. nidulans* hyphae (32). For each configuration, we compute the MFPT for capture at the microtubule plus ends both for distally initiated and uniformly initiated cargo (Fig. 4).

One of the key features of the microtubule configuration is the extent to which it covers the entire cellular domain. This is particularly important for the case of cargo entry at the distal tip, for which the presence of a microtubule end near the entry point can greatly speed up capture. We use the length of the longest microtubule in each configuration to describe this feature, demonstrating that the capture time generally decreases as the longest microtubule length is increased (Fig. 4 *a i*). The optimal configuration in this case involves microtubule plus ends scattered over ∼40% of the length of the cellular region, with several microtubules approaching near-maximal length. It should be noted that for the case of very long domains, the dependence on the longest microtubule length becomes an even stronger predictor of the capture time, with little variability among different configurations that have the same longest length (see Supporting materials and methods, Section S6).

When cargo is produced uniformly throughout the domain, the capture efficiency is not so well correlated with the length of the longest microtubule (Fig. 4 *a ii*). In this scenario, the optimal microtubule architecture exhibits a broad dispersion of the plus ends throughout the domain (Fig. 4 *b ii*), in keeping with the broad initial distribution of the cargo. We therefore sought to establish another metric that quantifies the extent to which plus ends are dispersed or clustered throughout the domain.

For cargo produced at the distal tip of the cell, the length of the longest microtubule determines the position of the nearest capture region relative to the point of cargo entry. For cargo initiated uniformly across all axial positions, we can define an analogous quantity that we term the minimal-distance clustering metric (*d*). Namely, for a given set of microtubule end positions, we compute the expected value of the distance between a point selected uniformly at random and the nearest microtubule end to that point (see details in Materials and methods). Because particles are also captured at the cell body, a capture region at *x* = 0 is appended to all microtubule configurations. The minimal-distance metric measures the clustering of capture regions: high values correspond to highly clustered microtubule plus ends (with *d*_maxlen_ = 0.25 for the configuration in which all plus ends are at the distal tip); low values correspond to plus ends spread evenly out over the entire domain (minimal value *d*_min_ = 1/(4*n*_MT_ + 2)).

The MFPT to capture at microtubule ends is plotted versus this clustering metric for each of the sampled microtubule configurations in Fig. 4 *c*. When particles start at the distal end of the domain, the capture times are largely insensitive to the clustering metric (Fig. 4 *c i*). The most optimal (lowest MFPT) configuration for cargo produced at the distal tip (*blue triangle*) has a moderately high clustering metric (*d* = 0.12), corresponding to slightly separated ends near the distal tip (see Fig. 4 *b i*), similar to the optimum found in Fig. 2.

By contrast, when cargo starts uniformly throughout the domain, lower clustering ensures that there is always a capture region close to the starting position of the particle, allowing for faster capture times (Fig. 4 *c ii*). The optimal configuration sampled for this scenario (*green square*, illustrated in Fig. 4 *b ii*) has a relatively low clustering metric of *d* = 0.049, close to the minimal possible value of this metric (*d*_min_ = 0.046 for *n*_MT_ = 5). This effect arises because clustered configurations near the cell tip require the dispersed cargo to diffuse over long distances through the cell before it can either reach the cell body or the plus ends located near the cell tip. On the other hand, evenly dispersed plus ends provide capture regions throughout the cell so that all cargos have a capture region nearby regardless of where they initiate.

It should be noted that not all combinations of maximal length and clustering metric are accessible. Namely, the highest clustering metrics require the microtubule lengths to be tightly clustered near very short or very long values and cannot be reached by configurations with intermediate microtubule lengths (see Supporting materials and methods, Section S5). This effect yields a bimodal distribution of MFPTs at high clustering metric, as observed in Fig. 4 *c ii*.

For both distally initiated and uniformly dispersed cargo, the overall features of optimal microtubule configurations are unaltered if we include an explicit retrograde transit time to get the overall MFPT to reach the cell body (see Supporting materials and methods, Section S7). This is unsurprising because from any given position along the tubule, diffusive transport to the cell body is much slower than active retrograde transport, so the optimal microtubule architecture is dominated primarily by the initial capture time.

**Figure 5:**
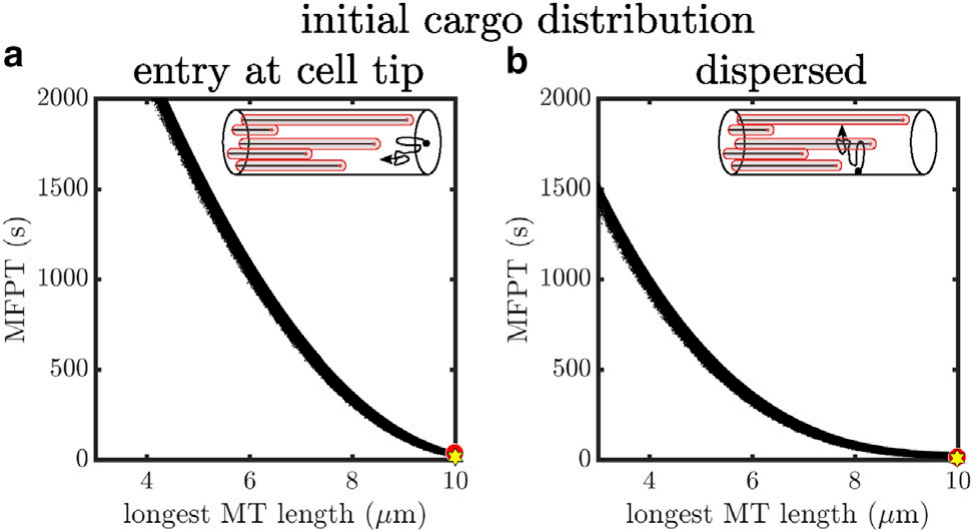
Cargo capture along full length of microtubule. Scatter plots of the MFPT for cargo capture along the full length of the microtubule (MT) are shown for 10^6^ randomly sampled configurations with five microtubules each in a domain of length 10 *μ*m. (*a*) Cargo is initiated at the distal tip. (*b*) Cargo is initiated uniformly throughout the domain. Yellow star denotes the configuration with the fastest capture for each initial cargo distribution. Red circle shows the globally optimized configuration corresponding to Fig. 4*b iii*. To see this figure in color, go online.

Notably, the results so far have focused on cargo that is captured by point-like dynein comets located at microtubule plus ends. However, dynein comets generally exhibit a gradual decrease in density over a micrometer length scale. In the Supporting materials and methods, Section S8, we provide equivalent results for the MFPT to capture by regions of increasing length. The extreme case corresponds to capture regions that are equal in length to the entire microtubule. Such a model is applicable to cargo that can be captured equally well along the full microtubule rather than just near the plus end. In Fig. 5, we see that in this situation the MFPT to capture is determined primarily by the length of the longest microtubule, regardless of whether the cargo is initiated distally or throughout the domain. This is a direct consequence of our assumption that the microtubules are nucleated near the cell body so that plus ends placed closer to the distal end correspond to a greater total length of microtubule available for capture.

Our analysis shows that the length of the longest microtubule is a strong predictor of the MFPT when cargos are captured by long regions of the microtubule (Fig. 5) and a moderate predictor when distally produced cargos are captured at the microtubule ends (Fig. 4 *a i*). In the case of uniformly dispersed cargo, the minimal-distance clustering metric is a complementary predictor of capture efficiency (Fig. 4 *c ii*). Microtubule configurations that fulfill both of these criteria (high longest length and low clustering) are expected to yield a fast capture time for all of the scenarios considered. We identify a set of six microtubule configurations that fall in the lowest 3% of MFPT for both distally produced and uniformly produced cargos and for the two extremes of plus-end capture and capture along the whole microtubule. Of these, the configuration with the lowest MFPT for distal initiation and plus-end capture is shown with red circles in Figs. 4 and 5. The configuration has one microtubule reaching nearly to the end of the domain and the other microtubules distributed roughly evenly over more than half of the domain length (Fig. 4 *b iii*).

Overall, these findings highlight the features of ideal microtubule configurations for efficiently capturing cargo for retrograde transport. Namely, configurations with one long microtubule and other microtubules of broadly distributed lengths result in near-optimal capture times regardless of whether cargos are produced distally or throughout the domain and of whether they are captured by point-like dynein comets at microtubule ends or more broadly along the whole microtubule.

### Establishing optimal configurations through microtubule dynamics

The results above demonstrate the overarching features of microtubule configurations that result in optimal cargo capture. Our model is agnostic as to the dynamic processes by which a cell might establish such an optimal configuration. Furthermore, a key simplifying assumption of the model is that individual microtubule architectures remain fixed throughout the capture process, so the distribution of microtubule lengths serves as a source of quenched disorder for the position of the capture regions. Realistically, microtubules in fungal hyphae grow and shrink on roughly 30 s timescales (44). Microtubules in growing neuronal projections are similarly dynamic, although those in mature axons tend to remain relatively stable over time (50,51). A variety of prior studies have highlighted the importance of microtubule dynamics in dictating the timescales of capture for relatively stationary cellular targets (including mitotic kinetochores and cortical regions) (52, 53, 54, 55). In this section, we briefly explore the role of plus-end dynamics in the capture of diffusive cargo.

We incorporate microtubule dynamics in the three-dimensional simulations by including basic growth and catastrophe processes, as described in Materials and methods, while fixing a total number of *n*_MT_ = 5 capture-capable microtubule tips. Our minimal dynamic microtubule model fixes the growth velocity (*v*_*g*_ = 0.18 *μ*m/s) according to published data in fungal hyphae (44). The catastrophe rate *k*_cat_ sets a timescale on which a growing microtubule halts and begins to shrink and is used as a free control parameter to tune microtubule distributions. Microtubules that reach the end of the domain are assumed to be capped and to remain fixed until a catastrophe event occurs.

**Figure 6:**
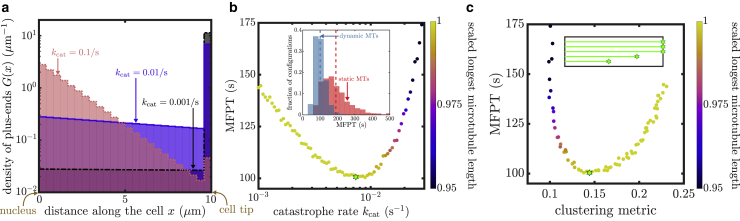
Cargo capture by dynamic microtubules. (*a*) Steady-state distributions of microtubule plus ends along the cell axis for different values of the catastrophe rate (*k*_cat_). (*b*) MFPT to capture cargo versus *k*_cat_ for dynamic microtubules. Inset compares the distribution of capture times to simulations with stationary microtubules sampled from the steady-state length distribution corresponding to the optimal value of *k*_cat_ (marked with *green star*). Dashed lines indicate the mean value for the corresponding distributions. (*c*) MFPT to capture versus average clustering (minimal-distance metric) for dynamic microtubules. The green star indicates the configuration with minimal MFPT. The inset denotes a representative microtubule configuration corresponding to the optimal catastrophe rate. Color in (*b*) and (*c*) indicates the average length of the longest microtubule, scaled by the domain length. All plots are shown for a domain of length *L* = 10 *μ*m, radius *r* = 1 *μ*m, and five dynamic microtubules with growth rate *v*_*g*_ = 0.18 *μ*m/s. MFPTs are obtained using three-dimensional simulations, with cargo starting at the distal tip. To see this figure in color, go online.

The catastrophe rate modulates the steady-state distribution of microtubule lengths (Fig. 6 *a*). In this simple model, the length of the longest microtubule in the domain and the clustering of microtubule ends are coupled together. Low values of *k*_cat_ result in most of the microtubule plus ends accumulating at the distal tip of the domain, corresponding to a high value for the longest microtubule length and for the clustering metric. Intermediate values of *k*_cat_ allow the microtubule ends to spread more broadly through the domain, whereas high values result in substantial shortening of all microtubules.

We carry out simulations with dynamic microtubules, focusing on the mean time to capture by microtubule plus ends for particles starting at the distal tip of the cell. Because of the coupling between the longest microtubule length and the end clustering, an optimal value of *k*_cat_ ≈ 0.007 s^−1^ emerges for minimizing capture time (Fig. 6 *b*). For this value, the longest microtubules are still able to reach the distal tip of the cell, but other microtubule ends remain relatively well scattered over a broad span of the distal region, as indicated by an average clustering metric of *d* ≈ 0.14 (Fig. 6 *c*). This optimal catastrophe rate is within the range of the measured values (0.006–0.04 s^−1^) in a variety of cellular systems (44). The existence of an optimal catastrophe rate of this order of magnitude has previously been established in quantitative models of “search and capture” of mitotic kinetochores by the plus ends of dynamically growing and shrinking microtubules (35,37).

We note that the absolute values for the capture times are substantially lower when microtubule dynamics are included in the simulation (Fig. 6 *b*, *inset*). This difference arises from a combination of two effects. First, growing microtubule ends sweep through the domain, tending to pick up any particles that have meandered away from the distal region. Second, the ability of dynamic microtubules to sample several configurations over the hundred-second timescale of particle capture makes it more likely that some microtubule end will encounter the particle, precluding the occasional very long trajectories associated with particles having to return to the distal end for capture. These results emphasize the importance of microtubule dynamics for efficient capture not only of stationary targets (37,52) but also of vesicular organelles destined for retrograde transport (36).

Despite the overall faster capture times, the dynamic model reproduces the overall features of optimal microtubule end configurations for particle capture. The existence of an optimal catastrophe rate further highlights the balance between allowing a few microtubules to stretch to the distal end of the cell while retaining a broad distribution of microtubule ends throughout the domain.

### Microtubule arrangements in *A. nidulans*

The theoretical work described here provides guiding principles for the performance of different microtubule architectures in capturing cargo. A logical avenue for further study would be to quantify microtubule configurations in actual cellular domains, and to compare the distributions observed with the features identified for optimal capture. To this end, we image hyphae of the fungus *A. nidulans* and visualize microtubule plus ends along the hyphal axis.

*A. nidulans* is a filamentous fungus that forms multinuclear tubular projections (hyphae). Owing to its genetic tractability and simplified geometry, *A. nidulans* has been used as a model organism for studies of microtubule-based transport (18,32,56). In the hyphal region beyond the most distal nucleus, microtubules form parallel, polarized arrangements, with plus ends growing toward the distal tip (18). The distal hyphal segment is on the order of 10 *μ*m in length and 1 *μ*m in radius (57,58), allowing it to be approximated as a narrow, effectively one-dimensional tubular region. Endosomes carrying signaling particles are thought to initiate primarily at the distal tip (59,60), whereas other organelles, such as peroxisomes, may form by fission or budding from the endoplasmic reticulum throughout the hyphal axis (61).

**Figure 7:**
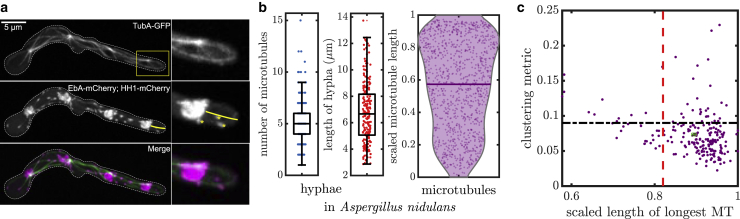
Microtubule configurations in *A. nidulans* hyphae. (*a*) Micrographs of *A. nidulans* germlings expressing fluorescently tagged tubulin (TubA-GFP, *top*), plus-end binding protein EbA/EB1 (EbA-mCherry), and nuclei (HH1-mCherry, *middle panel*). White dotted line shows the outline of the hypha. Yellow box denotes the cropped region shown on the right. Yellow line denotes measured length between hyphal tip and closest nucleus. Yellow asterisks denote EB1 plus ends. (*b*) Number of microtubules (*left*), hypha length (*center*), and scaled microtubule length (*right*) for *n* = 210 hyphal tip regions. Microtubule lengths are scaled by the length of the corresponding region from the last nucleus to the cell tip. (*c*) Scatter plot showing the scaled length of the longest microtubule and the clustering metric for hyphal microtubule configurations. The mean value for these metrics is indicated by the green point (scaled longest microtubule length 0.89 ± 0.005; clustering metric *d* = 0.073 ± 0.002). The vertical red line (0.82 ± 0.0002) and the horizontal black line (0.09 ± 0.00004) denote the average value of each corresponding metric for 10^6^ uniformly sampled configurations. All intervals and error bars correspond to mean ± SE. To see this figure in color, go online.

*A. nidulans* germlings (spores that have recently germinated to form hyphae) expressing GFP-tagged microtubules (tubulin TubA-GFP), mCherry-tagged microtubule plus ends (microtubule plus-end associated protein EB1 [EbA]-mCherry), and mCherry-tagged nuclei (histone H1 [HH1]-mCherry) were imaged using spinning disk confocal microscopy (details in the Supporting materials and methods, Section S9). Sections of the hypha extending from the last nucleus to the cell tip were chosen for analysis. Hyphal length from nucleus to tip was determined by tracing a line along the axis from the end of the last nucleus to the cell tip (Fig. 7 *a*, *left*). Microtubule plus ends were enumerated by counting EbA-mCherry puncta within the region beyond the last nucleus (Fig. 7 *a*, *right*). Finally, lengths of microtubules were estimated by projecting the locations of the EbA-mCherry puncta along the traced hyphal axis (*yellow line* in Fig. 7 *a*).

Based on data from *n* = 210 hyphae, the average length of the region from the last nucleus to the tip was 6.76 ± 0.16 *μ*m (mean ± standard error (SE)). Each postnuclear hypha region contained 5.13 ± 0.15 (mean ± SE) microtubules with an average length of 4.15 ± 0.08 *μ*m (mean ± SE). Fig. 7 *b* shows the distributions of the observed hypha and microtubule lengths. Because of the large variability in cell size, we scale microtubule lengths with respect to the length of the individual hypha. For all hyphae, scaled length of the longest microtubule and the minimal-distance clustering metric for microtubule plus-end positions are calculated and plotted in Fig. 7 *c*.

We compare microtubule arrangements in *A. nidulans* to the null hypothesis of microtubule ends scattered uniformly throughout the domain. This comparison helps identify nonuniform features of the microtubule distribution, which can then be compared to our computational predictions for optimal arrangements. To this end, we generate 10^6^ randomly sampled microtubule configurations. Each configuration has a number of microtubules drawn from the distribution observed in *A. nidulans* hyphae (Fig. 7 *b*, *left*), with each scaled microtubule length selected uniformly at random (between 0 and 1). The longest microtubule length and the clustering metric are computed for each random configuration, and the average values are plotted as dashed lines on Fig. 7 *c*.

The quantification of observed hyphal microtubule configurations demonstrated substantial differences from the null hypothesis of uniformly distributed random configurations. Namely, the mean scaled length of the longest microtubule was significantly longer than the value that would be expected for uniform architectures (*p* < 0.001 from one-sided *t*-test). Furthermore, the mean clustering metric for *A. nidulans* microtubules (0.073 ± 0.002) is significantly lower than the 0.09 ± 0.00004 value for random configurations (*p* < 0.001 from one-sided *t*-test). It should be noted, by contrast, that configurations selected specifically for long microtubules would be expected to have a clustering metric substantially above the uniformly distributed value because of the accumulation of multiple plus ends at the distal tip.

These comparisons indicate that microtubule arrangements in *A. nidulans* hyphae tend to have one long microtubule, with the remaining plus ends broadly distributed throughout the domain. These features match the optimal configuration predicted from the computational model for particle capture at plus ends. The hyphal measurements highlight the fact that microtubules exhibit a distinctly nonuniform, yet nonclustered, length distribution that should lead to efficient capture of both particles entering at the cell tip and those produced throughout the entire hypha.

## Conclusions

We have employed analytical modeling and computational simulations to highlight the role of microtubule arrangements in capturing cargo within tubular cells. For cargo entering at the distal end of the cell and captured at microtubule plus ends, we show that spreading capture regions away from the entry point results in faster engagement with microtubules. The effect of cell size on such optimal arrangements is explored, revealing that for cell lengths on the order of 10 *μ*m, it is optimal to distribute microtubule ends over up to 25% of the axial length. In longer cells, it becomes advantageous to cluster the plus ends over a relatively smaller fraction of the domain.

By analyzing random microtubule configurations, we establish general principles for rapid cargo capture across various scenarios for initial cargo distribution and capture modality. We show that configurations with a single long microtubule reaching the cell tip, accompanied by broad dispersal of the remaining microtubule ends, are ideal for rapidly capturing a variety of cargo. Such distributions can be established by tuning microtubule catastrophe rates as highlighted by simulations of cargo capture in a minimal model of dynamic microtubules. Notably, our results emphasize that an intermediate catastrophe rate is optimal for capture not just because it allows for more rapid microtubule dynamics (35,37) but also because it enables a broader steady-state distribution of microtubule plus ends.

Finally, we image microtubules in *A. nidulans* hyphae and show that their length distributions follow the general principles for optimality laid down by our model. These results highlight important aspects of cytoskeletal organization and its impact on cargo capture, providing possible mechanisms to establish optimal arrangements and validating predictions using in vivo data.

A central challenge for microtubule organization in a cell is the necessity for a single cytoskeletal architecture to serve as a transport highway for a variety of different cargos with different transport objectives. In this study, we focused specifically on the retrograde delivery of cargo to the nuclear region. However, other cargos require delivery from the nucleus to the periphery or broad distribution throughout the cellular domain. These alternate objectives impose different utility functions on the possible microtubule configurations. For directed delivery, long microtubules enable cargo to be deposited close to the distal tip, but axial separation of microtubule end positions has been shown to promote retention at the tip by reducing the recirculation of entrained cytoplasmic fluid (62). For broad distribution of bidirectionally moving cargo, short microtubules may reduce the processive run time of directed transport (41), resulting in increased frequency of reversals and more rapid distribution of cargo (12). Architectures with short microtubules of mixed polarity can also enable dispersion of cargos whose motion is dominated by a single motor type, as observed in the proximal regions of mammalian dendrites (63). The analytically tractable one-dimensional modeling approach and three-dimensional simulations developed here can be extended in future work to consider the impact of microtubule length distributions on this broad variety of intracellular transport systems.

By delineating the role of microtubule arrangements in individual transport processes, we can begin to gain a comprehensive picture of the evolutionary pressures guiding the observed microtubule architectures in live cells. Furthermore, establishing the impact of cytoskeletal morphology on the efficiency of key transport objectives is critical to developing a predictive understanding of how pharmacological or genetic perturbations in cytoskeletal filament length modulate cellular functions.

## Author contributions

S.S.M. and E.F.K. conceived and designed the research and developed the model. S.S.M. developed and implemented simulations and mathematical models, as well as analyzing imaging and simulation data. J.R.C. and S.L.R.-P. generated experimental data and performed imaging studies. All authors contributed to data interpretation and writing of the manuscript.

## Supporting citations

References (64, 65, 66, 67) appear in the Supporting material.

## Supporting material

Document S1. Supporting materials and methods, Figs. S1–S5, and Tables S1 and S2

Document S2. Article plus supporting material
