# Supplementary material for: Optimizing microtubule arrangements for rapid cargo capture
Source: Biophys J. 2021 Oct 21;120(22):4918–31. doi: 10.1016/j.bpj.2021.10.020 (PMC8633829; doi:10.1016/j.bpj.2021.10.020)
Supplement: Document S1. Supporting materials and methods, Figs. S1–S5, and Tables S1 and S2 [file mmc1.pdf]

Biophysical Journal, Volume *120*

Supplemental information

Optimizing microtubule arrangements for rapid cargo capture

Saurabh S. Mogre, Jenna R. Christensen, Samara L. Reck-Peterson, and Elena F. Koslover

Supplementary Material for
Optimizing microtubule arrangements for rapid cargo capture

S. S. Mogre, J. R. Christensen, S. L. Reck-Peterson, E. F. Koslover
Corresponding author: E. F. Koslover, ekoslover@ucsd.edu

S1. 1D MODEL FOR CALCULATING THE MFPT TO CAPTURE DIFFUSIVE CARGO

The 1D representation of capture regions for a particular distribution of microtubule lengths can be obtained as described in the Methods section of the main text. The cellular region is denoted by a linear domain of length L, with partially absorbing intervals positioned at the axial location of microtubule plus-ends. Assume that the 1D representation divides the domain into N intervals. Each interval has a fixed, discrete number of overlapping plus-end capture regions. Boundaries of each interval are denoted by the points x_i, $0 \leq i \leq N$, such that the ith interval is bounded by x_{i-1} and x_i. The first interval begins at the reflecting boundary $x_0 = L$ (cell tip), and the last interval ends at the perfectly absorbing boundary at $x_N = 0$ (cell body). The length of an interval is denoted as ℓ_{ik}, which is the length between the node x_i and x_k, where $k = i \pm 1$. The absorption rate in the intervals bounding the node x_i is similarly denoted as γ_{ik}, $k = i \pm 1$. The absorption rates γ_{ik} are linearly proportional to the number of microtubule plus-end capture regions that overlap on that interval. The rates are treated as integer multiples of the single-end absorption rate k_a.

For a particle that starts at node x_i, the splitting probability of diffusing to the neighboring nodes $x_{i\pm1}$ without being captured is given by

$$P_{ik} = \lim_{s\to0} \alpha_{ik} \left(\sinh \alpha_{ik}\ell_{ik} \sum_{j=i\pm1} \alpha_{ij} \coth \alpha_{ij}\ell_{ij} \right)^{-1}, \tag{S1}$$

where $\alpha_{ij} = \sqrt{(s + \gamma_{ij})/D}$, and $k = i \pm 1$. The duration for which the particle has remained within the intervals adjacent to x_i, and has not been absorbed or reached another node ($x_{i\pm1}$) is given by the waiting time

$$Q_i = \lim_{s\to0} \frac{1}{D} \frac{\sum_{j=i\pm1} \frac{1}{\alpha_{ij}} \tanh(\frac{\alpha_{ij}\ell_{ij}}{2})}{\sum_{j=i\pm1} \alpha_{ij} \coth(\alpha_{ij}\ell_{ij})}. \tag{S2}$$

The mean first passage time to capture for particles starting at the distal end can then be calculated as

$$\tau = \vec{V} \cdot (\mathbf{I} - \mathbf{P})^{-1} \cdot \vec{Q}, \tag{S3}$$

where $\mathbf{P}$ is an $N \times N$ matrix whose elements P_{ik} represent the splitting probabilities between nodes, with the rows and columns corresponding to the absorbing boundary at node x_{N+1} ($x = 0$) are set equal to zero. $\vec{Q}$ is an $N \times 1$ vector with elements representing the waiting time at each node except x_{N+1}. $\vec{V}$ is an $1 \times N$ vector denoting the initial particle distribution at each node (for distally produced particles: $V_0 = 1$ and all other elements are zero).

For cargo that starts uniformly distributed along an interval m, the splitting probability and the

waiting time to leave the interval at its bounding node x_j ($m-1 \leq j \leq m$) are given by

$$\begin{aligned} P_{mj}^{(E)} &= \lim_{s\to 0} \frac{1}{\alpha_m \ell_m} \tanh\left(\frac{\alpha_m \ell_m}{2}\right) \\ Q_m^{(E)} &= \lim_{s\to 0} \frac{1}{\alpha_m^2 D}\left[1 - \frac{2}{\alpha_m \ell_m}\tanh\left(\frac{\alpha_m \ell_m}{2}\right)\right]. \end{aligned} \tag{S4}$$

Eq. S3 can be modified to obtain the MFPT for cargo initially distributed uniformly throughout the cell as

$$\tau^{(E)} = \vec{W}^{(E)} \cdot \left[\vec{Q}^{(E)} + \mathbf{P}^{(E)} \cdot (\mathbf{I} - \mathbf{P})^{-1} \cdot \vec{Q}\right]. \tag{S5}$$

Here, $\vec{W}^{(E)}$ represents the initial distribution along each interval, and elements of $\vec{Q}^{(E)}$ and $\mathbf{P}^{(E)}$ are obtained using Eq. S4. Further details of the propagator based approach are available in Ref. [41].

S2. STEADY STATE DISTRIBUTION OF DYNAMIC MICROTUBULE PLUS-ENDS

Microtubule dynamics are incorporated using a basic model of growth and catastrophe as described in the methods section of the main text. Here, we obtain the steady-state probability density of microtubule plus-end positions $P(x)$ within the interval $0 \leq x \leq L$ representing the axis of the tubular cell. Microtubules are assumed to grow with velocity v_g within the linear interval, and enter a paused state upon reaching the cell tip at $x = L$. Both growing and paused microtubules can switch to a shrinking state with a catastrophe rate k_{cat}. Shrinking microtubules are assumed to instantaneously disappear, and are replaced by zero-length growing microtubules to maintain a constant number of capture-capable microtubules throughout the cell. Shrinking microtubules are assumed to have lost the dynein comet due to depolymerization, and thus are incapable of capture.

The dynamics of microtubule plus-end positions under these assumptions can be represented by

$$\begin{aligned} \frac{\partial}{\partial t} P(x,t) &= -v_g \frac{\partial}{\partial x} P(x,t) - k_{\text{cat}} P(x,t), \\ \frac{\partial}{\partial t} N_{\text{end}} &= v_g P(L,t) - k_{\text{cat}} N_{\text{end}}, \end{aligned} \tag{S6}$$

where the $P(x,t)$ is the density of growing microtubule ends and N_{end} is the number of microtubules paused at the distal tip. The boundary condition is given by setting the influx of growing microtubules at the cell body in such a way that the total number of microtubules ($n_{\text{MT}} = \int_0^L P(x) + N_{\text{end}} = 5$) stays constant. Integrating Eq. S6 over the domain and setting the resulting time derivative to zero yields the boundary condition:

$$v_g P(0,t) = k_{\text{cat}} n_{\text{MT}}$$

The steady-state solution for this system of equations is given by:

$$\begin{aligned} P(x) &= n_{\text{MT}} \left(\frac{k_{\text{cat}}}{v_g}\right) e^{-k_{\text{cat}} x / v_g}, \\ N_{\text{end}} &= n_{\text{MT}} e^{-k_{\text{cat}} L / v_g} \end{aligned} \tag{S7}$$

S3. LIST OF MODEL PARAMETERS

Parameter	Description	Value	Source
L	Length of tubular cellular region	10μm–100μm	This study
R	Radius of tubular cellular region	1μm	[32]
D	Diffusion coefficient of cargo	$0.01\mu\text{m}^2/\text{s}$	[24]
r	Radius of dynein comet	0.2μm	[20]
n_{MT}	Number of microtubules	5	This study
v_g	Growth velocity for dynamic microtubules	0.18μm/s	[42]

TABLE S1. Model parameters used in this study

S4. CAPTURE OF CARGO WITH A FINITE MATURATION RATE

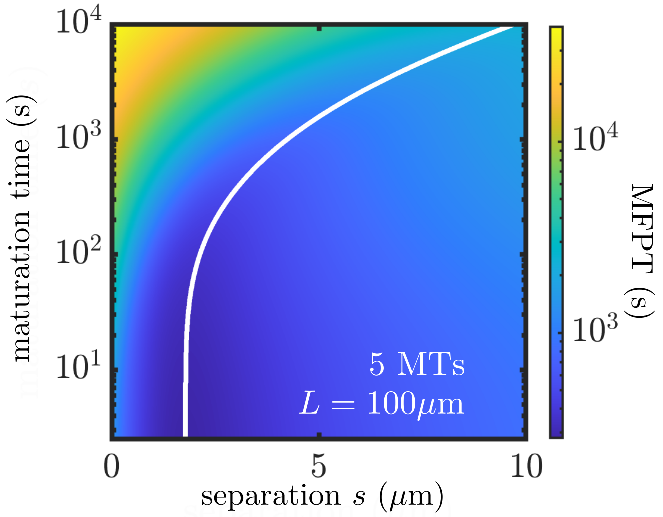

FIG. S1. **Capture times for maturing cargo.** MFPT for maturing cargos entering at the distal end of the cell and loaded at microtubule plus-ends, plotted against the maturation time and separation between consecutive microtubules. The white line denotes the separation with minimum MFPT for a given maturation time. Results are shown for a 100μm cellular region with 5 microtubules.

In some cases, the newly formed cargo may not be immediately available for capture, requiring additional maturation steps such as the acquisition of various adaptor proteins. We analyze optimal microtubule distributions for capture of cargo with a finite maturation time during which it moves diffusively without being able to bind to microtubule ends.

Varying the maturation rate effectively results in tuning the initial distribution of capture-ready cargo. For example, a very slow maturation rate results in a nearly uniform distribution of cargo available for capture since there is more time to diffuse before maturing. On the other hand, instantaneous maturation

reverts to the previously studied case of capture-ready cargo entering at the cell tip. Maturation of cargo can be incorporated as a constant-rate Poisson process that must be completed prior to capture, convolved together with a capture process where particles start in the appropriate distribution that spreads out from the distal end. Details of the calculation are described in prior work [41].

Fig. S1 shows the MFPT plotted against the maturation time and the separation between consecutive microtubules. As before, there is an optimum separation that minimizes the MFPT for each maturation time. The optimum separation increases as cargo maturation slows down, highlighting the need to spread microtubule plus-ends further in order to capture diffusively wandering cargo that matures slowly. For a 100μm cell, the optimal separation of microtubule tips begins to increase noticeably only for maturation times above 100 s.

S5. RELATION BETWEEN LENGTH OF LONGEST MICROTUBULE AND CLUSTERING METRIC

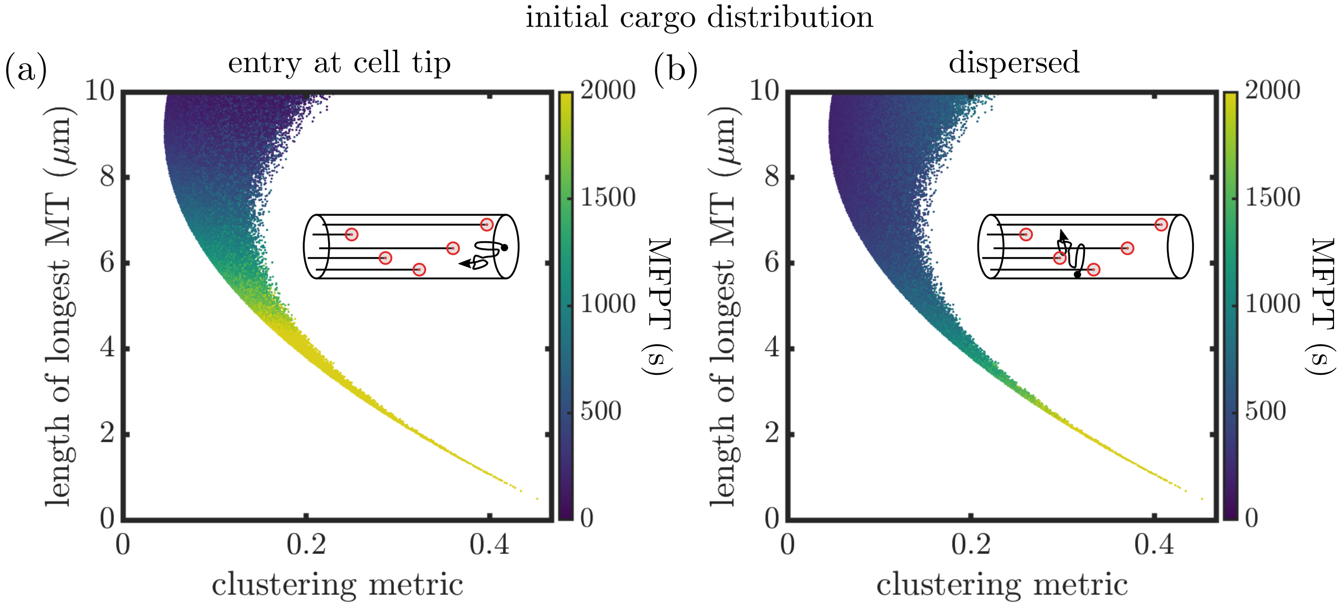

FIG. S2. **Some values of the clustering metric are inaccessible for intermediate lengths of longest microtubules.** Length of the longest microtubule, plotted against the clustering metric for 10^6 random configurations. The color indicates the MFPT to capture **(a)** cargo entering at the tip, and **(b)** cargo initially distributed uniformly.

The clustering metric d defined in the methods section of the main text provides a quantitative description of the dispersion of capture regions throughout the cell. In this section, we analyze the distribution of this clustering metric for a large set of microtubule configurations, focusing on the variation with the longest microtubule length. Our metric represents the average distance between a uniformly distributed probe and capture regions at microtubule plus-ends, along with an additional region at the cell body. The capture region at the cell body creates an inherent asymmetry in the metric with respect to the location of capture regions along the cell. Microtubule configurations with plus-ends clustered close to the cell body correspond to higher values of the clustering metric compared to configurations that are clustered near the tip of the cell (Fig. S2). An interesting consequence of this asymmetry is that certain values of d are inaccessible for configurations with intermediate length of the longest microtubule, which results

in a bimodal distribution of the MFPT for highly clustered configurations with the same value of the clustering metric. (See Fig. S3c, Fig. 4c)

S6. OPTIMAL CONFIGURATIONS FOR CARGO CAPTURE IN LONG CELLULAR REGIONS

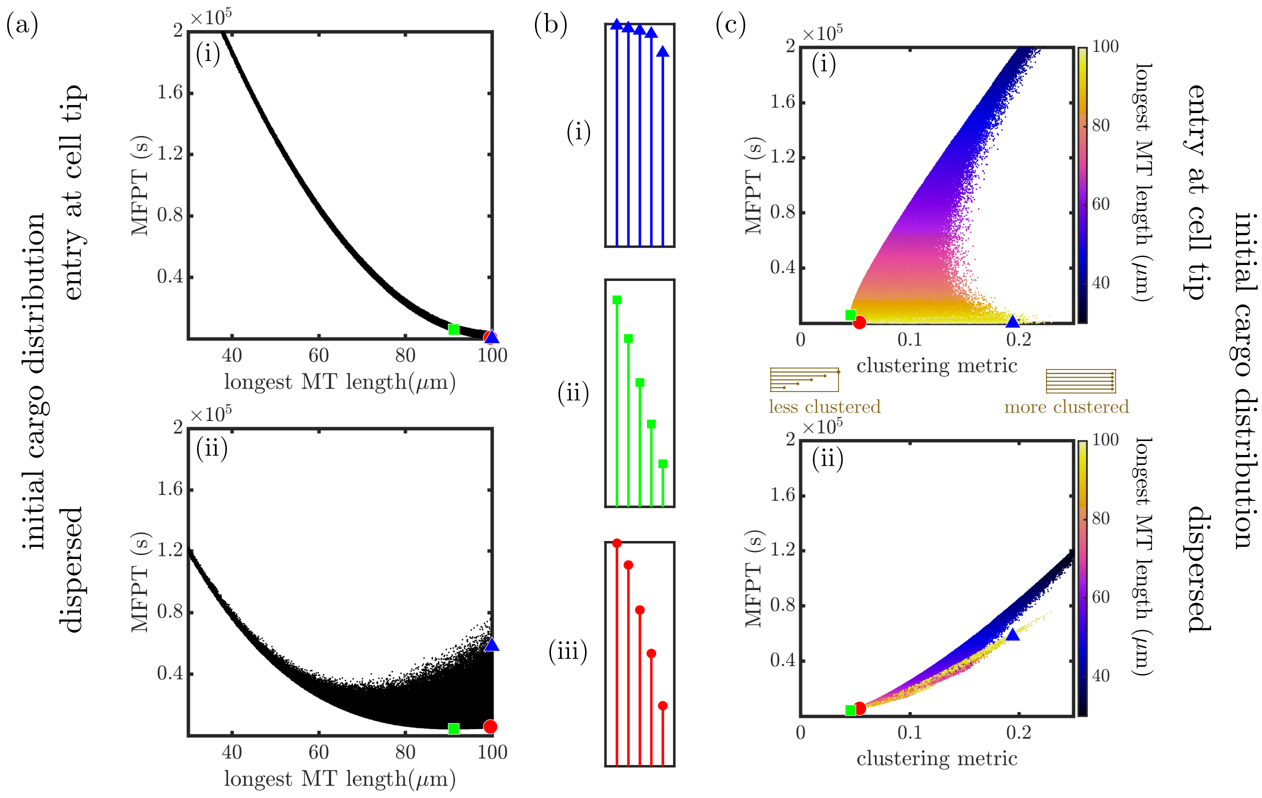

FIG. S3. **MFPT for random microtubule configurations**. (a) Scatter plots showing the MFPT to capture at microtubule plus-ends vs the length of the longest microtubule for 10^6 randomly sampled configurations with 5 microtubules each in a domain of length 100μm. (i) Cargos start at the cell tip. (ii) Cargos start uniformly. Blue triangle indicates the overall fastest configuration for (i). Green square indicates the overall fastest configuration for (ii). Red circle denotes a configuration that falls within the lowest 2.5% of MFPTs for both starting distributions. (b) Microtubule configurations corresponding to the (i) blue triangle, (ii) green square, and (iii) red circle in panel (a). (c) Scatter plots showing the MFPT plotted against a clustering metric for the randomly sampled configurations, with color indicating longest microtubule length for each configuration. (i) cargos start at cell tip. (ii) cargos start uniformly. Blue triangle, green square, and red circle denote configurations illustrated in panel (b).

Tubular regions can vary broadly in length, from 10μm hyphal tips, to axonal regions that can be orders of magnitude longer. Here, we repeat the calculations for Fig. 4, which identify the key features of optimal microtubule configurations, for a longer domain of length 100μm. For these longer domains, the longest microtubule length is a very strong predictor of the capture time for cargo entering at the cell tip. For cargo that is initially uniformly dispersed, the clustering metric forms a better predictor of the MFPT. Thus, longer domains amplify the effects seen in the 10μm domains considered in the main text.

It should be noted that the yellow dots in Fig. S3c.ii correspond to configurations where at least one microtubule stretches across the full domain. When the clustering metric is low and other tips are spread throughout the domain, these configurations give similar MFPTs regardless of the maximal length. When

the clustering metric is high, a clear separation is observed between configurations where all MT tips are the distal end and ones where all are clustered elsewhere in the domain. The former have much lower MFPTs than the latter, because clustered tips away from the distal region imply a substantial section of the domain is left uncovered by microtubules so that cargos formed there must diffuse a long distance to be captured. A similar separation of MFPTs at high clustering metrics (between clusters at the distal tip and clusters elsewhere) is seen in Fig. 4c.ii.

S7. RETROGRADE CARGO DELIVERY TO THE CELL BODY

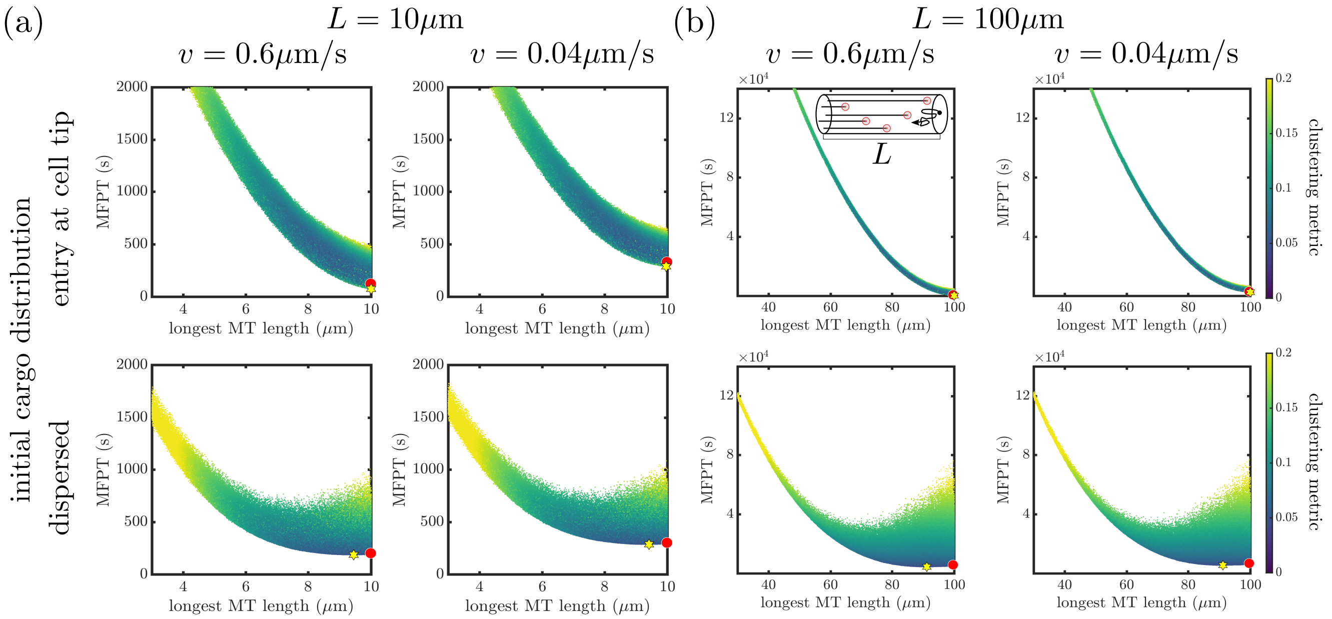

FIG. S4. **MFPT to deliver cargo to the cell body.** **(a)** Scatter plots showing the MFPT to reach the cell body (including the time to capture at microtubule plus-ends) vs the length of the longest microtubule for 10^6 randomly sampled configurations with 5 microtubules each in a domain of length 10μm. The cargo moves persistently towards the cell body with an effective velocity of $v = 0.6\mu$m/s (left), or $v = 0.04\mu$m/s (right) after being loaded onto the microtubule. Top: cargos start at the cell tip. Bottom: cargos start uniformly. The red circle denotes the configuration shown in Fig. 4b.iii of the main text. **(b)** Same plot as (a) for a cellular region of length 100μm. The red circle here denotes the configuration shown in Fig. S3b.iii. For both (a) and (b), yellow stars denote the overall fastest configuration for a given capture condition. The color of the scatter points denotes the clustering metric described in the main text.

The results shown in the main text focus on the role of microtubule length distribution in the initial loading of cargo, neglecting the time required to deliver the captured cargo to the cell body. Here, we further explore the extent to which incorporating retrograde transport itself alters the optimal microtubule configurations. While many organelles are observed to exhibit bidirectional motion along microtubules [20, 63], others (such as neuronal autophagosomes [47] and signaling endosomes [14]) move processively towards the cell body. Our focus here is on optimizing the specific cellular objective of retrograde transport (shortest time to reach the cell body). Assuming that the cargo does not dissociate back to a diffusive state after it is loaded onto the microtubules, the retrograde transport process can then be treated as an overall effective 'drift velocity' towards the cell body.

The retrograde transport time after capture can range widely depending on the length of the domain and the pausing or reversal behavior. We consider here two examples: particles with retrograde veloc-

ities of 0.6μm/s and 0.04μm/s, corresponding to measured values of effective average velocity towards the cell body for largely processive autophagosomes and bidirectional Rab5-marked early endosomes in hippocampal axons [64]. We also consider two domain lengths: $L = 10\mu$m and $L = 100\mu$m.

The time required to deliver cargo at a constant velocity in the retrograde direction can be incorporated in the MFPT calculations from Section S1. The probability of cargo being captured (loaded on a microtubule) in each absorbing region is given by

$$\vec{p}_{\text{abs}} = (\mathbf{I} - \mathbf{P}) \cdot \mathbb{1}, \tag{S8}$$

where $\mathbf{P}$ can be obtained using Eq. S1, and $\mathbb{1}$ is an appropriately sized column vector with all elements 1. Once the cargo is captured within an absorbing region, the time required to transport it to the cell body at a constant effective velocity v is given by

$$\vec{t}_{\text{move}} = \vec{y}/v, \tag{S9}$$

where $\vec{y}$ is a vector whose elements denote the distance of the midpoint of each absorbing region from the cell body. For the coordinates established in Sec. S1, the corresponding distance for interval i is given by $y_i = L - (x_i + x_{i-1})/2$. Here, we have assumed that the length of the absorbing region itself is small compared to its distance from the cell body. This assumption allows us to approximate the capture location as the midpoint of an absorbing region. For cargo captured at microtubule plus-ends in fungal hyphae, the size of an absorbing region is in the order of 0.4μm, while the distance from the cell body is in the order of 10μm, supporting the validity of the assumption for this particular system.

The overall time to deliver cargo to the cell body can be obtained by a weighted sum over all absorbing regions. The delivery time can be incorporated as an additional term in the survival time vector $\vec{Q}$. The MFPT for a cargo to reach the cell body can then be given by

$$\tau^{(CB)} = \vec{V} \cdot (\mathbf{I} - \mathbf{P})^{-1} \cdot \vec{Q}^{(CB)}, \tag{S10}$$

where

$$\vec{Q}^{(CB)} = \vec{Q} + (\vec{p}_{\text{abs}} \cdot \mathbb{1}^T) \cdot \vec{t}_{\text{move}}. \tag{S11}$$

Fig. S4 reproduces Fig. 4a and Fig. S3a, while incorporating the time required to travel to the cell body for two different domain lengths and effective retrograde velocities. We see that the qualitative results regarding optimal architectures for retrograde delivery still hold. For cargo originating at the cell tip, the MFPT is determined primarily by the length of the longest microtubule – an effect that is even more pronounced for longer domains. For dispersed cargo, optimal configurations have somewhat shorter maximum microtubule length and very low scores of the clustering metric. The general architecture indicated by the red dot, which has one maximally long microtubule and all other plus-ends broadly dispersed, falls within the lowest 2.5% of the calculated MSDs in each of the cases studied here. Thus, incorporation of processive retrograde movement does not substantially alter the optimal microtubule tip distribution within the range of parameters studied here.

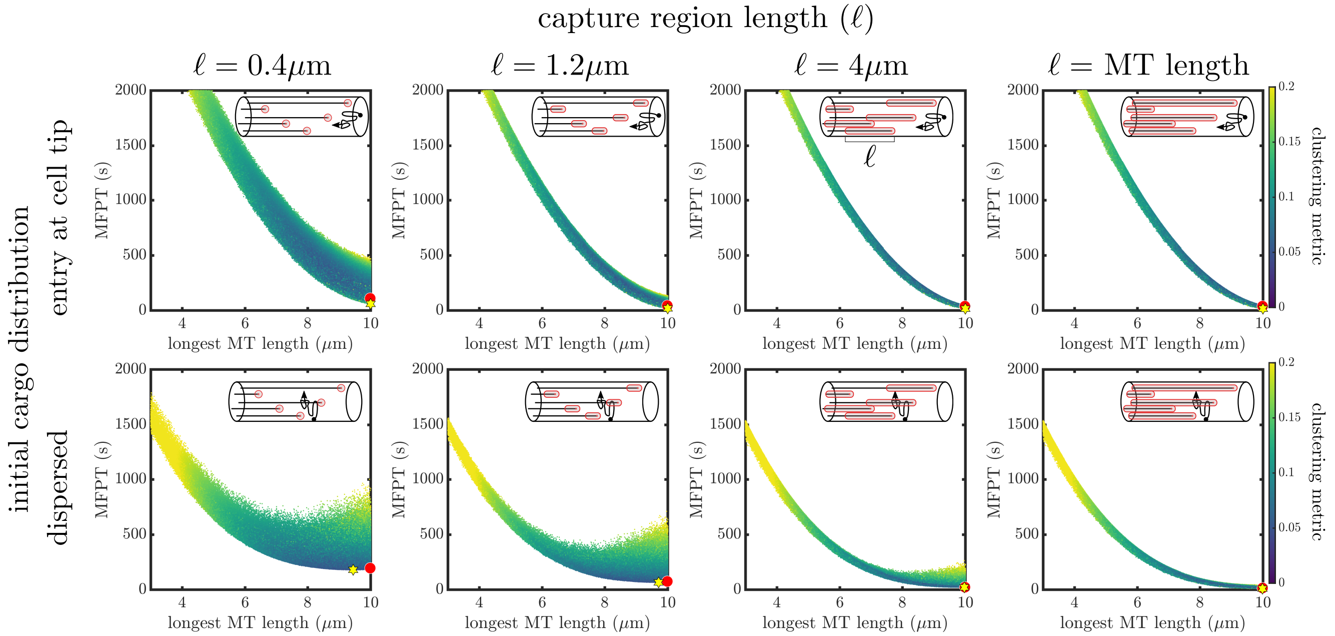

FIG. S5. **Cargo capture by regions of varying size.** Scatter plots showing the MFPT vs the length of the longest microtubule for 10^6 randomly sampled configurations with 5 microtubules each in a domain of length 10μm. The length of the capture region is indicated by ℓ. The top row denotes the MFPT for cargo formed at the cell tip. The bottom row denotes the MFPT for cargo initially dispersed uniformly. The red circle denotes a configuration that falls within the lowest 3% of MFPTs for both starting distributions for capture near plus-ends ($\ell = 0.4\mu$m, main text Fig. 4b.iii) and for capture along the whole microtubule (main text Fig. 5). Yellow stars denote the overall fastest configuration for a given capture condition. The color of the scatter points denotes the clustering metric described in the main text.

S8. EFFECT OF CAPTURE REGION SIZE ON CARGO CAPTURE TIME

In the main text, we focused on cargo loaded onto microtubules only within a 200nm contact radius of the plus-end. However, dynein comets generally exhibit a gradual decrease in density over a micrometer length scale [20]. Furthermore, some cargos may recruit their own motor protein assembly and should be able to initiate retrograde transport elsewhere along the microtubule. For a given arrangement of microtubule lengths, the cargo capture time can vary widely depending on the size and availability of regions along the microtubule where the cargo can bind. In this section, we quantify the relation between the size of the capture region along a microtubule and the MFPT to capture cargo.

Fig. S5 shows the MFPT for 10^6 randomly sampled configurations of 5 microtubules, for several different values of the capture region length ℓ (equivalent to twice the capture radius). The left-most plots correspond to Fig. 4a. The right-most plots represent the limiting case where cargo can be captured along the entire microtubule, corresponding to Fig. 5. In this limiting case, we see that the longest-microtubule length is a strong predictor of the capture time, with very little variation among the MFPT for different configurations with the same longest length. Intermediate values of the capture length behave essentially as an interpolation between narrow capture at the tip and capture along the whole microtubule.

The red dots in Fig. S5 correspond to the configuration shown in Fig. 4b.iii, which performs nearly optimally for plus-end capture with both cargo entering at the cell tip and cargo starting with a uniform distribution. This configuration has a single microtubule stretching all the way to the distal tip, with the other microtubule ends spaced out evenly throughout the domain. Notably, such a configuration also falls within the lowest 3% of MFPTs for the case with capture along the entire microtubule. Thus microtubule

architectures with these dual features are near-optimal for rapid initiation of retrograde transport in a broad variety of scenarios, including different cargo entry points and different lengths of microtubule capture regions.

S9. METHODS FOR GROWING AND IMAGING *ASPERGILLUS NIDULANS* STRAINS USED IN THIS STUDY

Aspergillus nidulans strains were grown on yeast extract and glucose media agar gum plates for maintenance [65]. For spinning disk microscopy of *A. nidulans* germlings, *A. nidulans* spores were resuspended in 1 mL of 0.01% Tween-80. The spore/Tween-80 solution was then added 1:1000 to 1% glucose minimal media with no supplements in a 4-chamber 35mm dish with #1.5 coverglass bottom (Cellvis), and incubated for 16-20 hours at 30°C. Germlings were imaged using a Yokogawa W1 confocal scanhead mounted to a Nikon Ti2 microscope with an Apo TIRF 100x 1.49 NA objective. The scope was run with NIS Elements using the 488nm and 561nm lines of a six-line (405nm, 445nm, 488nm, 515nm, 561nm, and 640nm) LUN-F-XL laser engine and a Prime95B camera (Photometrics). Image channels in 488 and 561 were acquired sequentially using bandpass filters for each channel (525/50 and 595/50). The 488nm laser was 0.740 mW measured at the objective, and the 561nm laser was 0.980 mW measured at the objective, with an exposure time of 200 milliseconds for each. Z-stacks were acquired using a piezo Z stage (Mad City Labs). As the germlings do not grow flat along the coverglass surface but frequently extend from the surface, the z-range used to image a field of germlings was set differently for different fields depending on germling extension from the coverglass surface.

For both identification of EbA/EB1 puncta in *A. nidulans* hyphal tips and for the images shown in Figure 6A, maximum intensity projections were generated from z-stacks in FIJI [66]. To count the number of microtubules in a hyphal tip, the number of microtubule plus-ends (identified by the presence of EbA/EB1) were counted in germlings in which the entire hyphal tip was included within the maximum intensity projection. Using the multi-point tool and ROI manager in FIJI, bright EbA/EB1 puncta between the hyphal tip and the first nucleus were identified and counted. All EbA/EB1 spots were overlaid with the TubA-GFP/microtubule channel to ensure that they corresponded to a microtubule end. Hyphal tip lengths were measured by manually tracing the hyphal axis from the last nucleus to the furthest point on the hyphal tip in FIJI. Microtubule lengths were measured by first drawing a line along the hyphal axis, along which all microtubule length measurements were taken. The edge of the nucleus closest to the hyphal tip was then identified and denoted as site x=0 along the hyphal axis. Any EbA/EB1 puncta located within the region between the edge of the nucleus and the hyphal tip was then identified, and its position in x along the hyphal axis determined (position in y, perpendicular to the hyphal axis, was ignored for the purposes of this measurement). The distance between the edge of the nucleus (x=0) and each EbA/EB1 puncta (x=#) was then measured.

Aspergillus nidulans strains used in this study are listed in Table I. Strain RPA361 expressing EbA-mCherry, TubA-GFP, and HH1-mCherry was created through genetic crossing, as previously described [67].

**Strain**	**Genotype**	**Source**
RPA361	*[ebA*-mCherry-*Afribo]*, *[tubA*-GFP-*Afpyro]*; *[HH1*-mCherry-*AfPyrG]*; *riboB2*; *pyroA4*; *pyrG89*; Δ*nkuA::bar*	This study

TABLE S2. *A. nidulans* strain used in this study
